# Ovarian response and outcomes of *in vitro* fertilization cycles in transgender men: Report of three cases with a live birth

**DOI:** 10.5935/1518-0557.20240114

**Published:** 2025

**Authors:** Laura Maria Almeida Maia, Leci Veiga Caetano Amorim, Cassia Maria Avelar, Victoria Furquim Werneck Marinho, Erica Becker de Sousa Xavier, Aline R. Lorenzon, Ricardo Mello Marinho, João Pedro Junqueira Caetano

**Affiliations:** 1 Huntington Pró-Criar Medicina Reprodutiva – Eugin Group, Belo Horizonte, MG, Brazil; 2 Hospital Mater Dei, Belo Horizonte, MG, Brazil.; 3 Huntington Medicina Reprodutiva – Eugin Group – São Paulo, SP, Brazil; 4 Faculdade de Ciências Médicas de Minas Gerais, Belo Horizonte, MG, Brazil

**Keywords:** transgender reproduction, assisted reproduction in trans men and cis women couple, transgender *in vitro* fertilization reproduction

## Abstract

*In vitro* fertilization (IVF) treatments have broadened to cover a variety of scenarios, including those involving transgender individuals or couples who wish to become parents, posing new challenges for the field of reproductive medicine. While there are limited reports of successful IVF births involving transgender men, concerns remain regarding the impact of long-term testosterone use on ovarian function, oocyte and embryo quality and IVF reproductive outcomes. This study details a case series of three trans men and cis women couples at a fertility clinic, resulting in one live birth. The couples underwent IVF with frozen blastocyst-stage embryos transferred to the cis women partners. The trans men, aged 32, 35, and 38, received ovarian stimulation with gonadotropins and dydrogesterone for pituitary suppression, and a GnRH agonist for final oocyte maturation. All had been on 250 mg of testosterone and paused treatment to resume menstrual cycles before IVF. Eggs were fertilized with donor sperm, and despite a lower-than-expected response to stimulation after short-term testosterone discontinuation, high-quality blastocysts were produced, leading to two pregnancies. Given the uncertainty regarding the long-term effects of testosterone on overall fertility, patients should be advised to consider cryopreservation before starting gender-affirming hormone therapy. Our case reports contribute to the knowledge in this area, highlighting the feasibility of oocyte collection, embryo development, pregnancies, and births in these individuals even after prolonged use of testosterone.

## INTRODUCTION

Assisted reproduction techniques, originally developed for addressing infertility in heterosexual couples, have expanded to encompass diverse scenarios including female and male same-sex couples, as well as single individuals, utilizing donated gametes or surrogacy when necessary. Recently, individuals or couples where one or both partners are transgender have expressed a desire for children, presenting a new challenge for reproductive medicine. Although the response to ovarian stimulation of young trans-gender men is lower than expected, it is possible to obtain good quality blastocysts and pregnancies (Adeleye *et al.,* 2019).

Gender dysphoria, characterized by a mismatch between genetic sex and gender identity, affects approximately 0.5% of the population (de Ziegler & de Sutter, 2021). Recent data indicates an estimated 750,000 transgender individuals in Brazil – IBGE, 2018 (Pirtea *et al.,* 2021).

Gender-affirming hormone therapy (GAHT) for trans-gender men involves prolonged exogenous testosterone administration to promote secondary male sexual characteristics and regression of female characteristics in adults, with pubertal blockade for prepubertal individuals (Coleman *et al.,* 2012). Progestogens may also be used to suppress menstruation and provide contraception (Hembree *et al.,* 2017).

Ovarian stimulation for in vitro fertilization (IVF) cycles or oocyte cryopreservation can be emotionally challenging for transgender men due to the use of medications that increase estradiol production, serial vaginal ultrasound examinations and vaginal follicle aspiration. Few studies have evaluated the prolonged use of testosterone on ovarian function, oocyte quality, and fertility. Consequently, many authors recommend discontinuing testosterone before ovarian stimulation, although consensus on the optimal duration is still unknown (Albar *et al.,* 2023; Amir *et al.,* 2020; Greenwald *et al.,* 2021).

We present three cases reports involving IVF cycles, with ovarian stimulation, oocyte retrieval in transgender men and embryo transfer to their cisgender female partners. We analyzed ovarian response and the evolution of oocytes and embryos in the laboratory. Two clinical pregnancies were confirmed, and one live birth was achieved.

## CASE REPORTS

All couples provided informed consent for the publication of their cases.

### Case 1

A 32-year-old transgender man and a 34-year-old cisgender woman sought our assisted reproduction clinic for conception in February 2021. The transgender man had been using 250 mg intramuscular testosterone (Durateston®, Aspen Pharma, South Africa) for seven years and had undergone bilateral mastectomy six years prior. He discontinued testosterone therapy and awaited the resumption of menstrual cycles to start the IVF cycle, which resumed approximately two months post-consultation. Initial ultrasound revealed 11 antral follicles.

**Controlled Ovarian Stimulation (COS) and Egg Retrieval:** COS involved hMG (human menopausal go-nadotropin, Menopur®, Ferring Pharmaceuticals, Switzerland) with an initial dose of 300 IU for the first two days, reduced to 225 IU on the third day, and further reduced to 150 IU from the sixth to ninth day, totaling 1875 IU. Pituitary suppression was achieved with daily 20mg progestin (Duphaston®, Abbott Laboratories, USA) throughout COS. Triggering was performed with a GnRH agonist (Gonapeptyl® Daily, Ferring Pharmaceuticals) when follicles reached appropriate size. Oocyte retrieval was performed in April 2021, yielding seven oocytes (five matures, MII and two immatures, MI), resulting in three 2PN zygotes after ICSI. Two blastocysts (day 5, 4AB; day 6, 5BC, morphological grading according to Gardner *et al.* (2000), were cryopreserved.

**Embryo Transfer (ET):** The cisgender female partner underwent endometrial preparation with 6 mg/day oral estradiol valerate (Primogyna®, Bayer, Germany) for 12 days, achieving an endometrial thickness of 8.7 mm in May 2021. Luteal phase support was initiated with micronized vaginal progesterone 800 mg/day (Utrogestan®, Besins Healthcare, Monaco). Initial β-hCG (beta human chorionic gonadotropin) levels were 93.8 mIU/mL at nine days post-transfer and 231.8 mIU/L two days later. A first trimester ultrasound showed a gestational sac, but the patient subsequently experienced a miscarriage and underwent uterine curettage.

### Case 2

A 35-year-old transgender man and a 41-year-old cisgender woman presented at our private assisted reproduction clinic seeking assistance to conceive in March 2021. The transgender man had been on 250 mg injectable testosterone (Durateston®) for eight years and had undergone bilateral mastectomy six years prior. He discontinued testosterone therapy three months before ovarian stimulation and was advised to wait for the return of menstrual cycles. Initial transvaginal ultrasound showed 4 antral follicles.

**COS and Egg Retrieval:** COS was performed with hMG (Menopur®) starting with 225 IU daily for four days, followed by 300 IU daily for the next seven days, totaling 3000 IU. Pituitary suppression was achieved with 20 mg oral progestin (Duphaston®) daily from the start of COS. Follicular growth was monitored using transvaginal ultrasound. Trigger was performed with hCG (10000 IU) (Choriomon®-M; Meizler, Brazil) and a GnRH agonist (1 ampoule of Gonapeptyl® Daily) when at least three follicles reached a mean diameter of 17 mm. Oocyte retrieval was conducted 35 hours post-trigger, resulting in the retrieval of five oocytes (four MII and one MI) in June 2021. Following intracytoplasmic sperm injection (ICSI) with donor sperm, three 2PN zygotes were obtained. One high-quality blastocyst (day 5 3AA, Gardner criteria) (Gardner *et al.,* 2000) was cryopreserved.

**ET:** The cisgender female partner underwent endometrial preparation with 6 mg/day oral estradiol valerate (Primogyna®) for 14 days until achieving an endometrial thickness of 9.3 mm in November 2021. Luteal phase support was initiated with micronized vaginal progesterone 800 mg/day (Utrogestan®). A single blastocyst transfer resulted in a positive pregnancy test (serum β-hCG 202 mIU/L) nine days post-transfer, confirmed by ultrasound at 14 days. The pregnancy progressed without complications, culminating in a vaginal delivery at 39 weeks and 6 days. The newborn weighed 3200 grams, with no reported abnormalities.

### Case 3

A 38-year-old transgender man and a 36-year-old cisgender woman presented at our clinic for IVF treatment in April 2023. The transgender man had used 250 mg intramuscular testosterone (Durateston®) for six years, discontinuing its use two years prior in order to perform an ART treatment in another service. Hormonal anti-mullerian hormone (AMH) levels were 0.75 ng/mL, and initial transvaginal ultrasound showed 11 antral follicles.

**COS and Egg Retrieval:** COS utilized recombinant human gonadotropin (r-hFSH/r-hLH; Pergoveris®, Merck KGaA, Germany) starting with 300 IU for the first two days, reduced to 225 IU on the third day, and further reduced to 150 IU from the eighth to ninth day, totaling 2025 IU. Pituitary suppression was achieved with daily 20 mg progestin (Duphaston®) throughout COS. Triggering was performed with a GnRH agonist (Gonapeptyl® Daily) when follicles reached appropriate size. Oocyte retrieval, in July 2023, yielded fifteen oocytes (all MII), resulting in thirteen 2PN zygotes after ICSI. Six high-quality blastocysts were produced, with one transferred fresh (day 5 5AA) and five cryopreserved (three embryo day 5 5 AA, one day 5 4AA and one day 5 4AB, according to Gardner criteria) (Gardner *et al.* , 2000).

**ET:** The cisgender female partner underwent endometrial preparation with 6 mg/day oral estradiol valerate (Primogyna®) for nine days, achieving an endometrial thickness of 8.0 mm in July 2023. Luteal phase support was initiated with micronized vaginal progesterone 800 mg/day (Utrogestan®). Fresh embryo transfer resulted in a negative β-hCG (2.0 mIU/mL) at nine days post-transfer. Until the present moment, the couple have not returned seeking the transfer of the cryopreserved embryos.

## DISCUSSION

The American Society for Reproductive Medicine (ASRM) and the European Society of Human Reproduction and Embryology (ESHRE) assert that transgender patients should have equal access to fertility options as cisgender patients, and fertility preservation discussions ideally should occur before gender transition (De Wert *et al.,* 2014; Ethics Committee of the American Society for Reproductive Medicine, 2015; Ethics Committee of the American Society for Reproductive Medicine, 2021). A recent report indicates that 50% of transgender men surveyed prior to gender-affirming hormone therapy expressed a desire for children (Resende *et al.,* 2020), and 76% had considered fertility preservation before transitioning (Auer *et al.,* 2018).

Pregnancy in transgender individuals may lead to unconventional social role outcomes (e.g., reverse of traditional parental roles based on gamete involvement). However, these arguments do not justify denying these options to transgender individuals (Murphy, 2012).

Oocyte preservation is currently the primary option, although it can be costly and involves temporary suspension of testosterone therapy, use of gonadotropins, and frequent monitoring that may exacerbate gender dysphoria (Martin *et al.,* 2021; Pirtea *et al.,* 2021). Embryo cryopreservation is generally not chosen unless there is a male partner. Ovarian tissue cryopreservation presents a promising alternative, as it does not require hormonal therapy and tissue can be obtained during gender-affirming surgery. While successful oocyte retrieval post-ovarian tissue reimplantation has been reported, in vitro oocyte maturation remains experimental. Therefore, ovarian tissue cryopreservation would necessitate retransplantation with restoration of female endocrinology, which may be undesired for transgender men (Leung *et al.,* 2019).

In cases where oocyte cryopreservation was not performed previously, transgender men can undergo ovarian stimulation and in vitro fertilization with embryo transfer to the uterus of a cisgender female partner or a gestational carrier. In these cases, donor sperm is also required.

In our reported cases 1 and 2, testosterone use was discontinued until menstruation resumed, which occurred between 2 and 5 months, after which the cycle was initiated. In the third case, testosterone had been suspended for 2 years as the couple had received guidance at another clinic for treatment.

Cases 1 and 2 had suboptimal responses for their age, with Case 2 showing diminished ovarian reserve indicated by a low antral follicle count (AFC). In Case 3, despite a good number of antral follicles, the anti-Müllerian hormone (AMH) was low, but the response exceeded expectations, coinciding with the longest duration of testosterone suspension.

All cases resulted in the production of good-quality blastocysts. In Case 2, where only 1 blastocyst was obtained, this led to a full-term pregnancy and a healthy newborn. Case 1 experienced a miscarriage, while Case 3, with was the oldest patient (38 years old), had a negative beta hCG despite a better COS response.

Most published studies evaluating ovarian stimulation primarily involved cycles for oocyte freezing in transgender men before or after transition, including adolescents (Insogna *et al.,* 2020). There are also reports of successful IVF cycles, including in Brazil (Resende *et al.,* 2020; White *et al.,* 2023).

In a study exploring other treatment options, a group with four cases of IVF cycles showed varied ovarian responses, with retrieval ranging from 3 to 28 mature oocytes, blastocyst production and 2 pregnancies, demonstrating a viable treatment with good outcomes for this family formation (Ghofranian *et al.,* 2023). Leung *et al.* (2019) also reported 7 IVF cycles with embryo transfer, resulting in pregnancies and births, as a part of an interesting study on ovarian stimulation that included oocyte freezing and comparison with a cisgender women group.

Despite these reports, there are uncertainties about the long-term effects of testosterone on ovarian function, response to stimulation, oocytes and embryos quality, and IVF outcomes.

All studies were observational, with inconsistent testosterone regimens and serum levels achieved. The average duration of testosterone therapy in most studies was less than 4 years, with varying durations reported, and a free time of hormonal therapy before the start of the cycle. Our cases report prolonged use of testosterone, ranging from 6 to 8 years, which exceeds the average reported in the literature.

Some authors suggest a potentially beneficial effect of testosterone on ovarian response by creating a biochemical environment similar to that seen in patients with polycystic ovary syndrome. This may explain the higher number of mature oocytes obtained in a retrospective cohort of 26 young female-to-male transgender individuals compared to cisgender women. However, the total dose of gonadotropins used in this study was significantly higher in the transgender group. Testosterone was discontinued in all cases, with menstruation or testosterone levels returning to the normal female range before proceeding (Leung *et al.,* 2019).

Changes in the ovaries due to testosterone treatment are also complicated by reportedly high rates of polycystic ovary syndrome (PCOS) in transgender men even before hormone therapy (15%-58%) (Pirtea *et al.,* 2021). Reviews on the effects of testosterone on ovarian morphology show conflicting results, with some studies indicating changes like PCOS, while others refute this notion (Ikeda *et al.,* 2013).

Another study comparing fertility preservation methods (embryo and oocyte cryopreservation) in transgender men with and without testosterone exposure and cisgender women showed no significant differences in hormone levels, number of retrieved oocytes, or oocyte maturity rates among the groups. However, it was noted a trend in a lower number of blastocyst development with the longest exposure to testosterone (more than 7 years), suggesting a potential negative impact on oocyte quality and embryonic development (Amir *et al.,* 2020).

In line with these findings, a recent study on ovarian histology and morphology of cumulus-oocyte complexes in transgender men revealed normal follicular complex morphology similar to fertile cisgender women. Furthermore, the study reported a good *in vitro* maturation rate of approximately 34% in cumulus-oocyte complexes retrieved from transgender men, comparable to that seen in other studies (De Roo *et al.,* 2016; 2017).

Baillie’s recent study demonstrated that testosterone exposure in vivo reduces follicle growth activation and morphological health, leading to apparent DNA damage, with further deterioration after culture compared to controls (Bailie *et al.,* 2023).

A research group has proposed oocyte collection for in vitro maturation in transgender men during sexual reassignment surgery. While positive results have been reported, ovarian tissue oocytes matured in vitro show low developmental capacity in transgender men undergoing testosterone treatment (Lierman *et al.,* 2021).

One of the main concerns of the transgender population is the suspension of testosterone therapy for oocyte stimulation and retrieval, potentially resulting in the loss of secondary male characteristics such as body mass and hair, and the return of female characteristics like fat distribution and menstruation. Most studies recommend testosterone suspension for 3-6 months to allow for the return of menses before beginning the process. However, there are case reports where testosterone therapy was not suspended, yet successful ovarian stimulation and oocyte retrieval were achieved (Cho *et al.,* 2020; Gale *et al.,* 2021; Greenwald *et al.,* 2021; Moravek *et al.,* 2023). The main questions regarding maintaining testosterone therapy during controlled ovarian stimulation include oocyte quality, ovarian response, effects on offspring, and the risk of ovarian hyperstimulation syndrome (Moravek *et al.,* 2020). An alternative to alleviate the stress of assisted reproductive technology (ART) procedures could be conducting controlled ovarian stimulation without waiting for menses, considering follicular waves during the ovarian cycle (Moravek *et al.,* 2023). Another interesting option to mitigate the adverse effects of estrogen exposure in patients undergoing fertility preservation could be the use of letrozole (Martin *et al.,* 2021; Stark & Mok-Lin, 2022).

## CONCLUSION

Current assisted reproduction techniques offer possibilities for transgender individuals to build families. However, further research is needed in this field, as many questions remain unanswered regarding fertility preservation for transgender men, including the long-term effects of testosterone therapy on ovarian function, oocyte quality, ideal timing for testosterone therapy suspension, and methods to minimize physical and psychological side effects of oocyte preservation treatments, thereby reducing gender dysphoria. It is crucial for healthcare providers responsible for transgender care to provide counseling on general health and fertility preservation. While many individuals express interest in future fertility, these options are not widely offered (Mattelin *et al.,* 2022).

Given the uncertainty regarding the long-term effects of testosterone on overall fertility, patients should be advised to consider cryopreservation before starting gender-affirming hormone therapy. Our case reports contribute to the knowledge in this area, highlighting the feasibility of oocyte collection, embryo development, pregnancies, and births in these individuals even after prolonged use of testosterone.
